# 2-Amino-4-ferrocenyl-5-oxo-5,6,7,8-tetra­hydro-4*H*-chromene-3-carbo­nitrile monohydrate

**DOI:** 10.1107/S2414314625004687

**Published:** 2025-05-30

**Authors:** Carren Nyapola, David O. Juma, Sizwe J. Zamisa, Eric M. Njogu, Bernard Omondi

**Affiliations:** a School of Chemistry and Physics, University of KwaZulu Natal, Private Bag X54001, Westville, Durban, 4000, South Africa; b Multimedia University of Kenya, PO Box 15653-00503, Nairobi, Kenya; Purdue University, USA

**Keywords:** crystal structure, ferrocen­yl, chromene

## Abstract

In the title hydrate, [Fe(C_5_H_5_)(C_15_H_13_N_2_O_2_)]·H_2_O, the pendent ferrocenyl substituent is significantly rotated against the chromene backbone, with a torsion angle of 56.8 (2)°. Rotational disorder is observed in one of the Cp rings of the ferrocenyl substituents. The crystal packing is consolidated by a network of O—H⋯N, O—H⋯O, N—H⋯O and N—H⋯π hydrogen bonds, prominently involving a solvent water mol­ecule. The water mol­ecule functions as both a hydrogen-bond donor and acceptor, bridging adjacent mol­ecules, leading to the formation of a layer with a distinctive hydrogen-bonded motif propagating parallel to the *bc* plane

## Abstract 2

The crystal packing is consolidated by O—H⋯N, O—H⋯O, N—H⋯O, and N—H⋯π hydrogen bonds, with the solvent water mol­ecule acting as both donor and acceptor. This results in a two-dimensional hydrogen-bonded network extending parallel to the *bc* plane.

## Graphical abstract

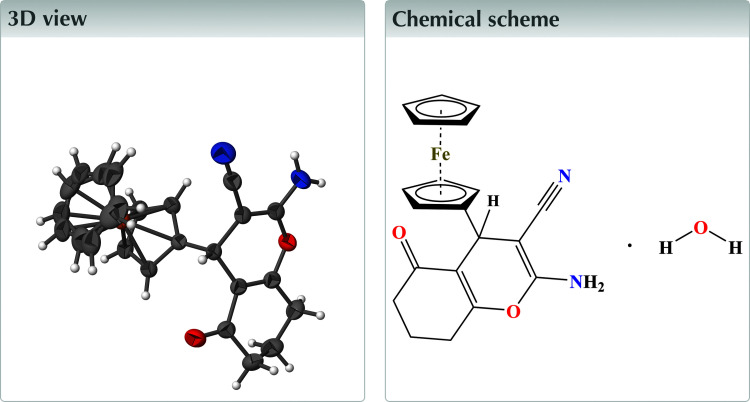

## Structure description

Recent pharmacological investigations have highlighted 2-amino-4*H*-pyran carbo­nitrile derivatives as promising anti­cancer agents, driven by their unique mol­ecular architecture and versatility (Mansouri *et al.*, 2011 ▸; Wang *et al.*, 2014 ▸, 2025 ▸). These compounds belong to the heterocyclic pyran family, renowned for diverse pharmacological applications ranging from anti­microbial to anti­tumor activities (Fouda, 2016 ▸; Kathrotiya & Patel, 2012 ▸; Veena *et al.*, 2022 ▸). Aryl-substituted 4*H*-chromene-3-carbo­nitriles exhibit strong DNA-binding affinities *via* hydrogen-bonding inter­actions at their amino groups, suggesting a mechanistic link to their biological activity (Zamisa *et al.*, 2022 ▸). Building upon the above findings, our recent work (Nyapola *et al.*, 2025 ▸) continues to expand the exploration of 4*H*-pyran derivatives for enhanced pharmacokinetic properties.

**Figure 1:**
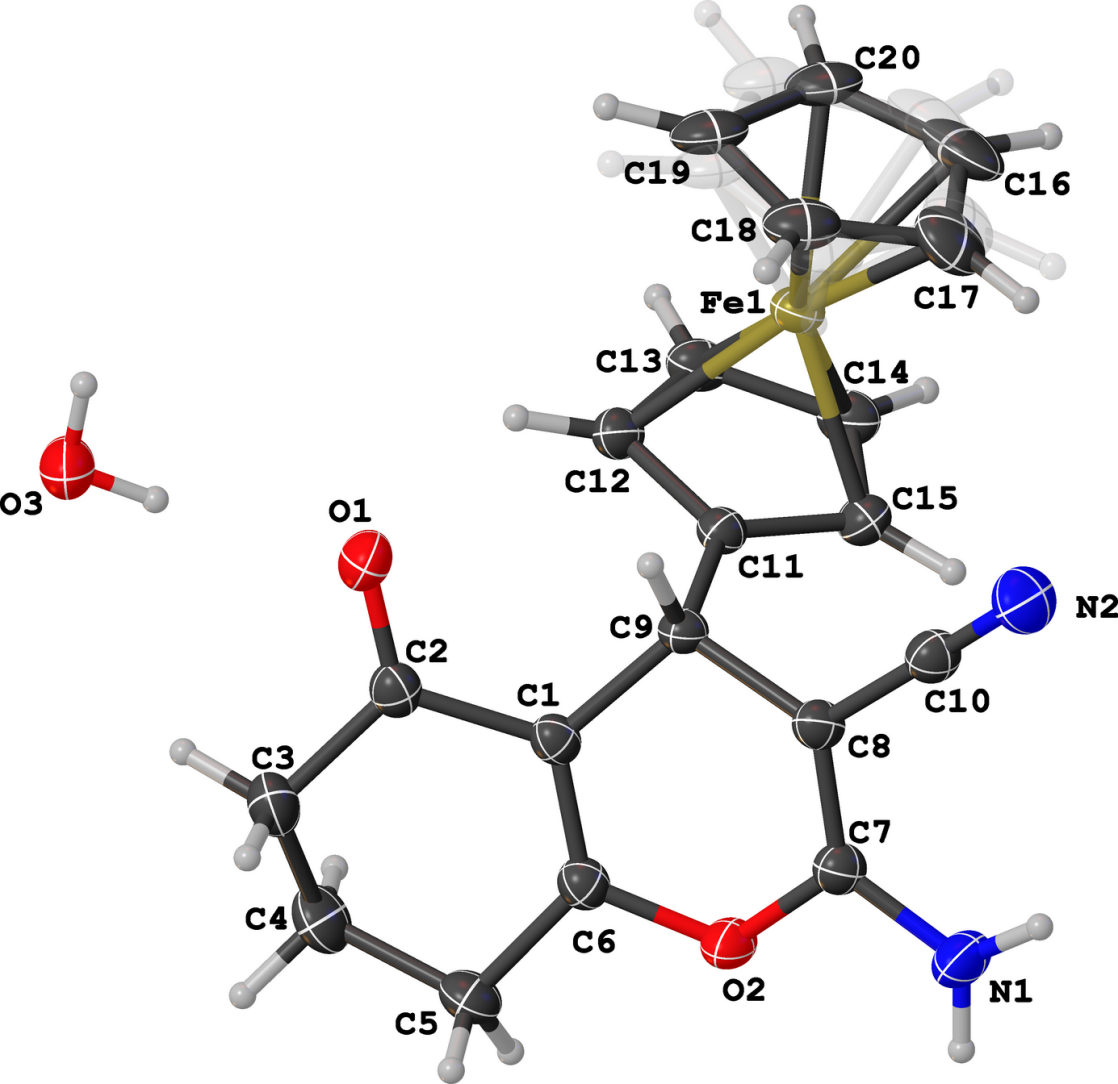
Mol­ecular structure of the title compound showing the atom-numbering scheme and displacement ellipsoids drawn at the 50% probability level. The minor disordered part of the Cp ring is given in a faint color.

**Figure 2:**
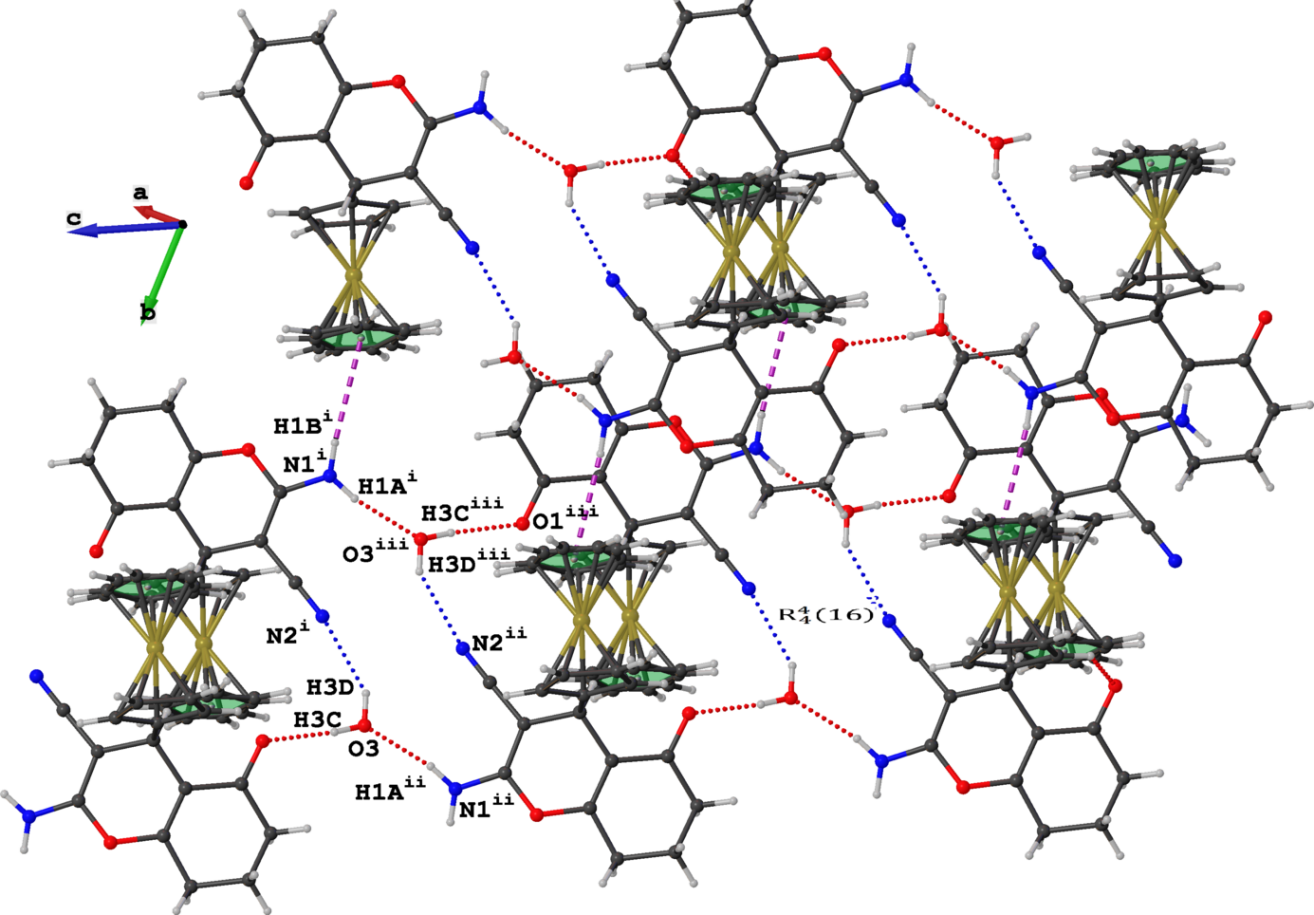
Hydrogen bonds in the crystal structure of the title compound. Symmetry codes: (i) −*x* + 2, −*y* + 1, −*z* + 1; (ii) *x*, *y*, *z* + 1; (iii) 2 − *x*, 1 − *y*, -*z.*

The mol­ecule of the title compound consists of a tetra­hydro­chromene moiety with ferrocenyl, cyano, amino, and oxo substituents, as shown in Fig. 1 ▸. The compound crystallizes in a centrosymmetric space group with one mol­ecule in the asymmetric unit. The pendent ferrocenyl substituent is significantly rotated against the chromene backbone, with a C1—C9—C11—C12 torsion angle of 56.8 (2)°. This is notably larger compared to the torsion angle of the pendant *p*-tolyl substituent in the closely related compound 2-amino-7,7-dimethyl-5-oxo-4-(*p*-tol­yl)-5,6,7,8-tetra­hydro-4*H*-chromene-3-carbo­nitrile (CSD ref code BOZMAI; Veeranagaiah *et al.*, 2025 ▸), where the torsion angle is 39.42 (14)°. The crystal structure of the title compound is consolidated by O—H⋯N, O—H⋯O, N—H⋯O and N—H⋯π hydrogen bonds (Table 1 ▸). The solvent water mol­ecule serves as a trifunctional hydrogen-bonding group, donating both of its hydrogen atoms to form O3—H3*C*⋯O1 and O3—H3*D*⋯N2 hydrogen bonds, thereby bridging two adjacent mol­ecules. Simultaneously, the oxygen atom of the water mol­ecule acts as a hydrogen-bond acceptor, participating in an N1—H1*A*⋯O3 inter­action, where the amine group donates one of its H atoms. The second amine H atom does not form a classical hydrogen bond but appears to form an N1—H1*B*⋯π inter­action towards the Cp ring C16–C20 at symmetry position *x*, 1 + *y*, *z*. Together, these inter­actions generate a supra­molecular layer structure featuring a characteristic hydrogen-bonded ring described by an 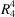
(16) graph-set motif, which extends parallel to the crystallographic *bc* plane (Fig. 2 ▸).

## Synthesis and crystallization

The title compound was synthesized *via* a one-pot reaction involving 1,3-cyclo­hexa­nedione (0.015 mmol), malono­nitrile (0.015 mmol), and ferrocene carboxaldehyde (0.015 mmol). Two drops of tri­ethyl­amine catalysed the reaction. Following the established synthetic procedure (Nyapola *et al.*, 2025 ▸), the reaction mixture was placed in a 35 ml snap-on microwave vessel and subjected to microwave irradiation at 100°C for 10 min. The reaction mixture was filtered off under vacuum and recrystallized from ethanol, yielding a light-green-coloured solid. Slow evaporation from acetone solution yielded single crystals.

## Refinement

Crystallographic data and structure refinement details are summarized in Table 2 ▸. The unsubstituted cyclo­penta­dienyl ring of the ferrocenyl substituent was refined as disordered over two positions. PART 1 and 2 instructions were used to model the disorder, and the major component site occupancy refined to a value of 0.515 (18). All disordered C—C bond lengths and C—C—C bond angles were restrained to be similar to each other (SADI restraints, e.s.d. 0.02 Å) and *U^ij^* components of ADPs for disordered atoms closer to each other than 2.0 Å were restrained to be similar (SIMU restraint, e.s.d. 0.01 Å^2^). Amine and water H-atom positions were refined and restrained to target values of 0.84 (1) and 0.86 (1) Å, respectively. *U*_iso_(H) values were set to a multiple of *U*_eq_(C/N/O) with 1.5 for water, and 1.2 for C—H, CH_2_, and NH_2_ units, respectively.

## Supplementary Material

Crystal structure: contains datablock(s) I. DOI: 10.1107/S2414314625004687/zl4082sup1.cif

Structure factors: contains datablock(s) I. DOI: 10.1107/S2414314625004687/zl4082Isup2.hkl

CCDC reference: 2453792

Additional supporting information:  crystallographic information; 3D view; checkCIF report

## Figures and Tables

**Table 1:** Hydrogen-bond geometry (Å, °) *Cg*1 and *Cg*2 are the centroids of the Cp rings C16–C20 and C16*A*–C20*A*, respectively.

*D*—H⋯*A*	*D*—H	H⋯*A*	*D*⋯*A*	*D*—H⋯*A*
O3—H3*C*⋯O1	0.84 (1)	2.01 (1)	2.842 (2)	175 (3)
O3—H3*D*⋯N2^i^	0.83 (1)	2.28 (2)	3.011 (3)	147 (3)
N1—H1*A*⋯O3^ii^	0.86 (1)	2.01 (1)	2.859 (3)	168 (3)
N1—H1*B*⋯*Cg*1^iii^	0.86 (2)	2.86 (2)	3.696 (7)	164 (3)
N1—H1*B*⋯*Cg*2^iii^	0.84 (2)	2.86 (2)	3.677 (7)	165 (3)

Symmetry codes: (i) 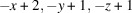; (ii) 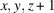; (iii) 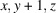.

**Table 2:** Experimental details

Crystal data	
Chemical formula	[Fe(C_5_H_5_)(C_15_H_13_N_2_O_2_)]·H_2_O
*M*_r_	392.23
Crystal system, space group	Triclinic, *P* 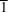
Temperature (K)	296
*a*, *b*, *c* (Å)	9.2440 (4), 10.3415 (4), 11.0093 (5)
α, β, γ (°)	65.358 (3), 66.714 (2), 81.236 (2)
*V* (Å^3^)	878.59 (7)
*Z*	2
Radiation type	Mo *K*α
μ (mm^−1^)	0.88
Crystal size (mm)	0.22 × 0.14 × 0.11

Data collection	
Diffractometer	Bruker APEXII CCD
Absorption correction	Multi-scan (*SADABS*; Krause *et al*., 2015 ▸)
*T*_min_, *T*_max_	0.456, 0.746
No. of measured, independent and observed [*I* > 2σ(*I*)] reflections	11443, 3810, 3248
*R*_int_	0.047
(sin θ/λ)_max_ (Å^−1^)	0.641

Refinement	
*R*[*F*^2^ > 2σ(*F*^2^)], *wR*(*F*^2^), *S*	0.039, 0.105, 1.06
No. of reflections	3810
No. of parameters	293
No. of restraints	244
H-atom treatment	H atoms treated by a mixture of independent and constrained refinement
Δρ_max_, Δρ_min_ (e Å^−3^)	0.61, −0.21

Computer programs: *APEX2* and *SAINT* (Bruker, 2009 ▸), *SHELXT2018/2* (Sheldrick, 2015*a* ▸), *SHELXL2019/3* (Sheldrick, 2015*b* ▸) and *OLEX2* (Dolomanov *et al.*, 2009 ▸).

## full crystallographic data

### 2-Amino-4-ferrocenyl-5-oxo-5,6,7,8-tetrahydro-4H-chromene-3-carbonitrile monohydrate Crystal data

**Table:**

[Fe(C_5_H_5_)(C_15_H_13_N_2_O_2_)]·H_2_O	*Z* = 2
*M**_r_* = 392.23	*F*(000) = 408
Triclinic, *P*1	*D*_x_ = 1.483 Mg m^−^^3^
*a* = 9.2440 (4) Å	Mo *K*α radiation, λ = 0.71073 Å
*b* = 10.3415 (4) Å	Cell parameters from 6839 reflections
*c* = 11.0093 (5) Å	θ = 2.4–28.0°
α = 65.358 (3)°	µ = 0.88 mm^−^^1^
β = 66.714 (2)°	*T* = 296 K
γ = 81.236 (2)°	Plate, orange
*V* = 878.59 (7) Å^3^	0.22 × 0.14 × 0.11 mm

### 2-Amino-4-ferrocenyl-5-oxo-5,6,7,8-tetrahydro-4H-chromene-3-carbonitrile monohydrate Data collection

**Table:**

Bruker APEXII CCD diffractometer	3810 independent reflections
Radiation source: microfocus sealed X-ray tube, Incoatec Iµs	3248 reflections with *I* > 2σ(*I*)
Graphite monochromator	*R*_int_ = 0.047
Detector resolution: 7.9 pixels mm^-1^	θ_max_ = 27.1°, θ_min_ = 2.2°
φ and ω scans	*h* = −11→11
Absorption correction: multi-scan (SADABS; Krause et al., 2015)	*k* = −13→11
*T*_min_ = 0.456, *T*_max_ = 0.746	*l* = −14→14
11443 measured reflections	

### 2-Amino-4-ferrocenyl-5-oxo-5,6,7,8-tetrahydro-4H-chromene-3-carbonitrile monohydrate Refinement

**Table:**

Refinement on *F*^2^	244 restraints
Least-squares matrix: full	Hydrogen site location: mixed
*R*[*F*^2^ > 2σ(*F*^2^)] = 0.039	H atoms treated by a mixture of independent and constrained refinement
*wR*(*F*^2^) = 0.105	*w* = 1/[σ^2^(*F*_o_^2^) + (0.0562*P*)^2^ + 0.1516*P*] where *P* = (*F*_o_^2^ + 2*F*_c_^2^)/3
*S* = 1.06	(Δ/σ)_max_ = 0.001
3810 reflections	Δρ_max_ = 0.61 e Å^−^^3^
293 parameters	Δρ_min_ = −0.21 e Å^−^^3^

### 2-Amino-4-ferrocenyl-5-oxo-5,6,7,8-tetrahydro-4H-chromene-3-carbonitrile monohydrate Special details

**Table:**

Geometry. All esds (except the esd in the dihedral angle between two l.s. planes) are estimated using the full covariance matrix. The cell esds are taken into account individually in the estimation of esds in distances, angles and torsion angles; correlations between esds in cell parameters are only used when they are defined by crystal symmetry. An approximate (isotropic) treatment of cell esds is used for estimating esds involving l.s. planes.

### 2-Amino-4-ferrocenyl-5-oxo-5,6,7,8-tetrahydro-4H-chromene-3-carbonitrile monohydrate Fractional atomic coordinates and isotropic or equivalent isotropic displacement parameters (Å^2^)

**Table:**

	*x*	*y*	*z*	*U*_iso_*/*U*_eq_	Occ. (<1)
Fe1	0.58891 (3)	0.41552 (3)	0.75251 (3)	0.03599 (12)	
O2	0.74860 (18)	0.98235 (15)	0.59337 (16)	0.0436 (3)	
O1	0.82245 (19)	0.74235 (17)	0.29140 (17)	0.0501 (4)	
O3	0.9227 (3)	0.7383 (2)	0.0134 (2)	0.0618 (5)	
H3C	0.894 (4)	0.745 (4)	0.093 (2)	0.093*	
H3D	0.957 (4)	0.6563 (18)	0.025 (4)	0.093*	
N1	0.8318 (3)	0.9127 (2)	0.7752 (2)	0.0525 (5)	
H1A	0.855 (3)	0.850 (2)	0.846 (2)	0.063*	
H1B	0.815 (3)	1.0006 (13)	0.763 (3)	0.063*	
C1	0.7747 (2)	0.8501 (2)	0.4537 (2)	0.0336 (4)	
C11	0.6289 (2)	0.63133 (19)	0.6557 (2)	0.0322 (4)	
C8	0.8416 (2)	0.7457 (2)	0.6690 (2)	0.0344 (4)	
N2	0.9740 (3)	0.5523 (2)	0.8274 (2)	0.0586 (5)	
C9	0.7864 (2)	0.71153 (19)	0.5728 (2)	0.0318 (4)	
H9	0.865531	0.652433	0.530185	0.038*	
C7	0.8102 (2)	0.8726 (2)	0.6821 (2)	0.0371 (4)	
C6	0.7517 (2)	0.9735 (2)	0.4709 (2)	0.0363 (4)	
C10	0.9149 (2)	0.6389 (2)	0.7575 (2)	0.0393 (4)	
C2	0.7964 (2)	0.8522 (2)	0.3127 (2)	0.0377 (4)	
C12	0.5419 (2)	0.5887 (2)	0.5956 (2)	0.0379 (4)	
H12	0.573472	0.602015	0.499779	0.046*	
C15	0.5366 (2)	0.5903 (2)	0.8046 (2)	0.0388 (4)	
H15	0.563734	0.605322	0.870646	0.047*	
C5	0.7248 (3)	1.1141 (2)	0.3676 (3)	0.0494 (5)	
H5A	0.819417	1.172602	0.320217	0.059*	
H5B	0.640441	1.161958	0.419066	0.059*	
C14	0.3966 (2)	0.5228 (2)	0.8357 (2)	0.0440 (5)	
H14	0.316893	0.485254	0.925717	0.053*	
C13	0.3984 (2)	0.5222 (2)	0.7081 (2)	0.0428 (5)	
H13	0.319911	0.484974	0.698423	0.051*	
C3	0.7901 (3)	0.9940 (3)	0.1965 (2)	0.0522 (6)	
H3A	0.754288	0.979939	0.131011	0.063*	
H3B	0.895323	1.035390	0.142229	0.063*	
C4	0.6816 (3)	1.0967 (3)	0.2555 (3)	0.0583 (6)	
H4A	0.573777	1.061783	0.298500	0.070*	
H4B	0.688090	1.188496	0.177336	0.070*	
C16	0.5873 (12)	0.2176 (11)	0.9122 (10)	0.0564 (19)	0.515 (18)
H16	0.518052	0.184267	1.007619	0.068*	0.515 (18)
C17	0.7345 (12)	0.2865 (13)	0.8579 (11)	0.0566 (17)	0.515 (18)
H17	0.778068	0.308042	0.910446	0.068*	0.515 (18)
C18	0.8026 (16)	0.316 (2)	0.7094 (13)	0.063 (3)	0.515 (18)
H18	0.900606	0.359344	0.647959	0.076*	0.515 (18)
C19	0.7011 (16)	0.2712 (12)	0.6684 (11)	0.0587 (19)	0.515 (18)
H19	0.718788	0.279958	0.576079	0.070*	0.515 (18)
C20	0.5634 (12)	0.2083 (9)	0.7957 (16)	0.0575 (18)	0.515 (18)
H20	0.475980	0.169245	0.800742	0.069*	0.515 (18)
C16A	0.6964 (16)	0.2675 (15)	0.8739 (11)	0.060 (2)	0.485 (18)
H16A	0.709339	0.270338	0.952386	0.072*	0.485 (18)
C17A	0.8021 (17)	0.325 (2)	0.7307 (14)	0.0545 (18)	0.485 (18)
H17A	0.895999	0.372651	0.697940	0.065*	0.485 (18)
C18A	0.7412 (13)	0.2961 (13)	0.6448 (11)	0.0548 (19)	0.485 (18)
H18A	0.789077	0.320784	0.546114	0.066*	0.485 (18)
C19A	0.5953 (12)	0.2240 (10)	0.7346 (16)	0.0526 (16)	0.485 (18)
H19A	0.529415	0.194251	0.705255	0.063*	0.485 (18)
C20A	0.5664 (13)	0.2048 (11)	0.8792 (13)	0.065 (3)	0.485 (18)
H20A	0.479224	0.159873	0.960754	0.079*	0.485 (18)

### 2-Amino-4-ferrocenyl-5-oxo-5,6,7,8-tetrahydro-4H-chromene-3-carbonitrile monohydrate Atomic displacement parameters (Å^2^)

**Table:**

	*U*^11^	*U*^22^	*U*^33^	*U*^12^	*U*^13^	*U*^23^
Fe1	0.04054 (18)	0.02748 (17)	0.0401 (2)	0.00073 (11)	−0.01778 (13)	−0.01079 (13)
O2	0.0592 (9)	0.0333 (7)	0.0486 (9)	0.0113 (6)	−0.0298 (7)	−0.0200 (7)
O1	0.0649 (10)	0.0500 (9)	0.0412 (9)	0.0029 (7)	−0.0219 (8)	−0.0219 (7)
O3	0.0961 (14)	0.0488 (10)	0.0463 (10)	0.0071 (9)	−0.0346 (10)	−0.0183 (9)
N1	0.0752 (13)	0.0449 (11)	0.0582 (13)	0.0106 (10)	−0.0415 (11)	−0.0269 (10)
C1	0.0327 (9)	0.0323 (10)	0.0345 (10)	−0.0002 (7)	−0.0145 (8)	−0.0097 (8)
C11	0.0360 (9)	0.0273 (9)	0.0350 (10)	0.0038 (7)	−0.0168 (8)	−0.0115 (8)
C8	0.0346 (9)	0.0322 (10)	0.0386 (11)	0.0012 (7)	−0.0176 (8)	−0.0124 (8)
N2	0.0692 (13)	0.0527 (12)	0.0652 (14)	0.0163 (10)	−0.0432 (12)	−0.0214 (11)
C9	0.0355 (9)	0.0271 (9)	0.0341 (10)	0.0020 (7)	−0.0142 (8)	−0.0123 (8)
C7	0.0385 (10)	0.0359 (10)	0.0405 (11)	0.0009 (8)	−0.0188 (9)	−0.0145 (9)
C6	0.0364 (10)	0.0345 (10)	0.0372 (11)	0.0019 (8)	−0.0158 (8)	−0.0117 (9)
C10	0.0402 (10)	0.0394 (11)	0.0437 (12)	0.0015 (8)	−0.0192 (9)	−0.0180 (9)
C2	0.0357 (10)	0.0404 (11)	0.0361 (11)	−0.0020 (8)	−0.0145 (8)	−0.0123 (9)
C12	0.0436 (11)	0.0345 (10)	0.0406 (11)	0.0020 (8)	−0.0223 (9)	−0.0133 (9)
C15	0.0445 (11)	0.0368 (11)	0.0385 (11)	0.0011 (8)	−0.0145 (9)	−0.0189 (9)
C5	0.0621 (14)	0.0311 (11)	0.0523 (14)	0.0066 (9)	−0.0264 (11)	−0.0110 (10)
C14	0.0380 (11)	0.0407 (11)	0.0470 (13)	−0.0012 (8)	−0.0079 (9)	−0.0180 (10)
C13	0.0399 (11)	0.0357 (11)	0.0557 (13)	0.0004 (8)	−0.0241 (10)	−0.0144 (10)
C3	0.0651 (15)	0.0489 (13)	0.0381 (12)	−0.0041 (11)	−0.0230 (11)	−0.0077 (10)
C4	0.0714 (16)	0.0488 (14)	0.0525 (15)	0.0096 (12)	−0.0359 (13)	−0.0084 (12)
C16	0.071 (3)	0.032 (3)	0.061 (3)	−0.003 (2)	−0.034 (3)	−0.004 (2)
C17	0.051 (3)	0.036 (3)	0.073 (3)	0.000 (2)	−0.036 (3)	0.000 (3)
C18	0.056 (3)	0.046 (3)	0.073 (4)	0.021 (3)	−0.020 (3)	−0.019 (3)
C19	0.072 (4)	0.034 (3)	0.071 (3)	0.008 (3)	−0.025 (3)	−0.026 (3)
C20	0.074 (3)	0.031 (2)	0.072 (4)	−0.002 (2)	−0.035 (3)	−0.016 (3)
C16A	0.068 (4)	0.046 (4)	0.060 (3)	0.008 (3)	−0.036 (3)	−0.006 (3)
C17A	0.057 (3)	0.039 (3)	0.073 (4)	0.011 (3)	−0.031 (3)	−0.024 (3)
C18A	0.057 (4)	0.036 (3)	0.070 (3)	0.006 (3)	−0.017 (3)	−0.027 (3)
C19A	0.067 (4)	0.028 (3)	0.068 (4)	0.004 (2)	−0.028 (3)	−0.023 (3)
C20A	0.075 (4)	0.032 (3)	0.067 (4)	−0.002 (3)	−0.023 (4)	0.000 (3)

### 2-Amino-4-ferrocenyl-5-oxo-5,6,7,8-tetrahydro-4H-chromene-3-carbonitrile monohydrate Geometric parameters (Å, º)

**Table:**

Fe1—C11	2.0518 (18)	C12—C13	1.426 (3)
Fe1—C12	2.039 (2)	C15—H15	0.9300
Fe1—C15	2.0518 (19)	C15—C14	1.416 (3)
Fe1—C14	2.047 (2)	C5—H5A	0.9700
Fe1—C13	2.049 (2)	C5—H5B	0.9700
Fe1—C18	2.057 (18)	C5—C4	1.521 (3)
Fe1—C19	2.021 (11)	C14—H14	0.9300
Fe1—C20	2.022 (8)	C14—C13	1.402 (3)
Fe1—C16A	1.999 (12)	C13—H13	0.9300
Fe1—C17A	2.019 (19)	C3—H3A	0.9700
Fe1—C19A	2.060 (8)	C3—H3B	0.9700
Fe1—C20A	2.028 (10)	C3—C4	1.513 (4)
O2—C7	1.373 (2)	C4—H4A	0.9700
O2—C6	1.378 (2)	C4—H4B	0.9700
O1—C2	1.224 (3)	C16—H16	0.9300
O3—H3C	0.836 (10)	C16—C17	1.419 (8)
O3—H3D	0.833 (10)	C16—C20	1.427 (9)
N1—H1A	0.864 (10)	C17—H17	0.9300
N1—H1B	0.859 (10)	C17—C18	1.411 (10)
N1—C7	1.345 (3)	C18—H18	0.9300
C1—C9	1.511 (3)	C18—C19	1.394 (10)
C1—C6	1.341 (3)	C19—H19	0.9300
C1—C2	1.475 (3)	C19—C20	1.445 (9)
C11—C9	1.526 (3)	C20—H20	0.9300
C11—C12	1.432 (3)	C16A—H16A	0.9300
C11—C15	1.421 (3)	C16A—C17A	1.404 (10)
C8—C9	1.517 (3)	C16A—C20A	1.421 (10)
C8—C7	1.354 (3)	C17A—H17A	0.9300
C8—C10	1.423 (3)	C17A—C18A	1.414 (11)
N2—C10	1.141 (3)	C18A—H18A	0.9300
C9—H9	0.9800	C18A—C19A	1.411 (8)
C6—C5	1.484 (3)	C19A—H19A	0.9300
C2—C3	1.503 (3)	C19A—C20A	1.436 (9)
C12—H12	0.9300	C20A—H20A	0.9300
			
C11—Fe1—C15	40.53 (8)	C11—C15—H15	125.7
C11—Fe1—C18	108.4 (4)	C14—C15—Fe1	69.60 (12)
C11—Fe1—C19A	149.3 (4)	C14—C15—C11	108.63 (18)
C12—Fe1—C11	40.99 (7)	C14—C15—H15	125.7
C12—Fe1—C15	68.07 (8)	C6—C5—H5A	109.5
C12—Fe1—C14	68.04 (9)	C6—C5—H5B	109.5
C12—Fe1—C13	40.85 (8)	C6—C5—C4	110.68 (19)
C12—Fe1—C18	120.9 (4)	H5A—C5—H5B	108.1
C12—Fe1—C19A	115.5 (4)	C4—C5—H5A	109.5
C15—Fe1—C18	126.8 (4)	C4—C5—H5B	109.5
C15—Fe1—C19A	167.7 (4)	Fe1—C14—H14	125.8
C14—Fe1—C11	68.43 (8)	C15—C14—Fe1	69.98 (11)
C14—Fe1—C15	40.43 (8)	C15—C14—H14	125.7
C14—Fe1—C13	40.03 (9)	C13—C14—Fe1	70.07 (12)
C14—Fe1—C18	163.5 (4)	C13—C14—C15	108.56 (19)
C14—Fe1—C19A	128.5 (3)	C13—C14—H14	125.7
C13—Fe1—C11	68.80 (8)	Fe1—C13—H13	126.4
C13—Fe1—C15	67.83 (8)	C12—C13—Fe1	69.19 (11)
C13—Fe1—C18	155.4 (4)	C12—C13—H13	126.1
C13—Fe1—C19A	106.7 (3)	C14—C13—Fe1	69.90 (12)
C19—Fe1—C11	126.5 (3)	C14—C13—C12	107.83 (18)
C19—Fe1—C12	108.5 (3)	C14—C13—H13	126.1
C19—Fe1—C15	163.6 (4)	C2—C3—H3A	109.1
C19—Fe1—C14	154.8 (4)	C2—C3—H3B	109.1
C19—Fe1—C13	120.8 (3)	C2—C3—C4	112.59 (19)
C19—Fe1—C18	40.0 (3)	H3A—C3—H3B	107.8
C19—Fe1—C20	41.9 (3)	C4—C3—H3A	109.1
C20—Fe1—C11	165.3 (4)	C4—C3—H3B	109.1
C20—Fe1—C12	127.3 (3)	C5—C4—H4A	109.4
C20—Fe1—C15	152.9 (4)	C5—C4—H4B	109.4
C20—Fe1—C14	118.9 (3)	C3—C4—C5	111.34 (19)
C20—Fe1—C13	107.9 (2)	C3—C4—H4A	109.4
C20—Fe1—C18	68.3 (5)	C3—C4—H4B	109.4
C16A—Fe1—C11	127.9 (4)	H4A—C4—H4B	108.0
C16A—Fe1—C12	163.9 (4)	Fe1—C16—H16	127.2
C16A—Fe1—C15	111.2 (4)	C17—C16—Fe1	70.6 (6)
C16A—Fe1—C14	122.6 (3)	C17—C16—H16	125.8
C16A—Fe1—C13	154.9 (4)	C17—C16—C20	108.4 (8)
C16A—Fe1—C17A	40.9 (4)	C20—C16—Fe1	67.9 (5)
C16A—Fe1—C19A	68.8 (4)	C20—C16—H16	125.8
C16A—Fe1—C20A	41.3 (3)	Fe1—C17—H17	126.7
C17A—Fe1—C11	106.4 (4)	C16—C17—Fe1	69.4 (6)
C17A—Fe1—C12	124.5 (4)	C16—C17—H17	126.4
C17A—Fe1—C15	120.3 (4)	C18—C17—Fe1	69.1 (9)
C17A—Fe1—C14	155.7 (4)	C18—C17—C16	107.3 (9)
C17A—Fe1—C13	162.3 (4)	C18—C17—H17	126.4
C17A—Fe1—C19A	68.3 (5)	Fe1—C18—H18	126.9
C17A—Fe1—C20A	69.1 (5)	C17—C18—Fe1	71.1 (8)
C20A—Fe1—C11	167.6 (5)	C17—C18—H18	125.0
C20A—Fe1—C12	151.1 (5)	C19—C18—Fe1	68.6 (8)
C20A—Fe1—C15	130.8 (4)	C19—C18—C17	109.9 (9)
C20A—Fe1—C14	110.6 (3)	C19—C18—H18	125.0
C20A—Fe1—C13	119.0 (4)	Fe1—C19—H19	124.8
C20A—Fe1—C19A	41.1 (3)	C18—C19—Fe1	71.4 (9)
C7—O2—C6	118.44 (15)	C18—C19—H19	126.3
H3C—O3—H3D	107 (3)	C18—C19—C20	107.5 (8)
H1A—N1—H1B	124 (3)	C20—C19—Fe1	69.1 (5)
C7—N1—H1A	119.5 (18)	C20—C19—H19	126.3
C7—N1—H1B	116.5 (18)	Fe1—C20—H20	124.8
C6—C1—C9	121.33 (18)	C16—C20—Fe1	71.2 (5)
C6—C1—C2	118.66 (18)	C16—C20—C19	106.9 (7)
C2—C1—C9	119.94 (17)	C16—C20—H20	126.6
C9—C11—Fe1	128.27 (12)	C19—C20—Fe1	69.0 (5)
C12—C11—Fe1	69.01 (11)	C19—C20—H20	126.6
C12—C11—C9	126.04 (17)	Fe1—C16A—H16A	125.2
C15—C11—Fe1	69.74 (11)	C17A—C16A—Fe1	70.3 (9)
C15—C11—C9	127.20 (17)	C17A—C16A—H16A	125.6
C15—C11—C12	106.71 (17)	C17A—C16A—C20A	108.7 (9)
C7—C8—C9	121.12 (17)	C20A—C16A—Fe1	70.4 (6)
C7—C8—C10	118.85 (18)	C20A—C16A—H16A	125.6
C10—C8—C9	119.78 (16)	Fe1—C17A—H17A	125.2
C1—C9—C11	110.74 (14)	C16A—C17A—Fe1	68.8 (8)
C1—C9—C8	107.86 (15)	C16A—C17A—H17A	125.9
C1—C9—H9	108.7	C16A—C17A—C18A	108.2 (9)
C11—C9—H9	108.7	C18A—C17A—Fe1	71.7 (8)
C8—C9—C11	112.10 (16)	C18A—C17A—H17A	125.9
C8—C9—H9	108.7	Fe1—C18A—H18A	128.2
N1—C7—O2	110.17 (17)	C17A—C18A—Fe1	67.9 (9)
N1—C7—C8	128.57 (19)	C17A—C18A—H18A	125.9
C8—C7—O2	121.25 (17)	C19A—C18A—Fe1	69.7 (5)
O2—C6—C5	111.49 (17)	C19A—C18A—C17A	108.3 (8)
C1—C6—O2	121.98 (18)	C19A—C18A—H18A	125.9
C1—C6—C5	126.52 (19)	Fe1—C19A—H19A	126.9
N2—C10—C8	179.2 (2)	C18A—C19A—Fe1	70.4 (5)
O1—C2—C1	120.63 (18)	C18A—C19A—H19A	126.0
O1—C2—C3	121.81 (19)	C18A—C19A—C20A	107.9 (8)
C1—C2—C3	117.53 (18)	C20A—C19A—Fe1	68.2 (5)
Fe1—C12—H12	125.8	C20A—C19A—H19A	126.0
C11—C12—Fe1	70.00 (11)	Fe1—C20A—H20A	126.1
C11—C12—H12	125.9	C16A—C20A—Fe1	68.3 (6)
C13—C12—Fe1	69.96 (12)	C16A—C20A—C19A	106.9 (8)
C13—C12—C11	108.27 (18)	C16A—C20A—H20A	126.6
C13—C12—H12	125.9	C19A—C20A—Fe1	70.7 (5)
Fe1—C15—H15	126.6	C19A—C20A—H20A	126.6
C11—C15—Fe1	69.73 (11)		
			
Fe1—C11—C9—C1	147.34 (14)	C6—C1—C2—O1	177.79 (19)
Fe1—C11—C9—C8	−92.15 (18)	C6—C1—C2—C3	−0.4 (3)
Fe1—C11—C12—C13	59.72 (14)	C6—C5—C4—C3	−46.1 (3)
Fe1—C11—C15—C14	−58.89 (14)	C10—C8—C9—C1	−158.07 (17)
Fe1—C12—C13—C14	59.45 (15)	C10—C8—C9—C11	79.8 (2)
Fe1—C15—C14—C13	−59.66 (15)	C10—C8—C7—O2	175.93 (18)
Fe1—C14—C13—C12	−59.00 (14)	C10—C8—C7—N1	−3.7 (3)
Fe1—C16—C17—C18	−58.9 (11)	C2—C1—C9—C11	−85.2 (2)
Fe1—C16—C20—C19	60.1 (6)	C2—C1—C9—C8	151.80 (16)
Fe1—C17—C18—C19	−57.7 (12)	C2—C1—C6—O2	−172.39 (17)
Fe1—C18—C19—C20	−60.0 (7)	C2—C1—C6—C5	8.5 (3)
Fe1—C19—C20—C16	−61.5 (7)	C2—C3—C4—C5	54.5 (3)
Fe1—C16A—C17A—C18A	−61.2 (13)	C12—C11—C9—C1	56.8 (2)
Fe1—C16A—C20A—C19A	60.4 (7)	C12—C11—C9—C8	177.28 (17)
Fe1—C17A—C18A—C19A	−58.0 (8)	C12—C11—C15—Fe1	59.38 (13)
Fe1—C18A—C19A—C20A	−58.1 (7)	C12—C11—C15—C14	0.5 (2)
Fe1—C19A—C20A—C16A	−58.9 (8)	C15—C11—C9—C1	−120.1 (2)
O2—C6—C5—C4	−163.64 (19)	C15—C11—C9—C8	0.4 (3)
O1—C2—C3—C4	150.8 (2)	C15—C11—C12—Fe1	−59.84 (13)
C1—C6—C5—C4	15.6 (3)	C15—C11—C12—C13	−0.1 (2)
C1—C2—C3—C4	−31.0 (3)	C15—C14—C13—Fe1	59.60 (15)
C11—C12—C13—Fe1	−59.74 (13)	C15—C14—C13—C12	0.6 (2)
C11—C12—C13—C14	−0.3 (2)	C16—C17—C18—Fe1	59.1 (8)
C11—C15—C14—Fe1	58.97 (14)	C16—C17—C18—C19	1.4 (18)
C11—C15—C14—C13	−0.7 (2)	C17—C16—C20—Fe1	−59.2 (8)
C9—C1—C6—O2	4.7 (3)	C17—C16—C20—C19	0.9 (11)
C9—C1—C6—C5	−174.49 (19)	C17—C18—C19—Fe1	59.2 (12)
C9—C1—C2—O1	0.7 (3)	C17—C18—C19—C20	−0.8 (17)
C9—C1—C2—C3	−177.52 (18)	C18—C19—C20—Fe1	61.4 (10)
C9—C11—C12—Fe1	122.77 (17)	C18—C19—C20—C16	−0.1 (13)
C9—C11—C12—C13	−177.51 (17)	C20—C16—C17—Fe1	57.5 (7)
C9—C11—C15—Fe1	−123.27 (18)	C20—C16—C17—C18	−1.4 (14)
C9—C11—C15—C14	177.84 (17)	C16A—C17A—C18A—Fe1	59.4 (12)
C9—C8—C7—O2	−9.9 (3)	C16A—C17A—C18A—C19A	1.3 (18)
C9—C8—C7—N1	170.5 (2)	C17A—C16A—C20A—Fe1	−60.1 (12)
C7—O2—C6—C1	17.1 (3)	C17A—C16A—C20A—C19A	0.3 (15)
C7—O2—C6—C5	−163.63 (18)	C17A—C18A—C19A—Fe1	56.9 (11)
C7—C8—C9—C1	27.8 (2)	C17A—C18A—C19A—C20A	−1.1 (14)
C7—C8—C9—C11	−94.4 (2)	C18A—C19A—C20A—Fe1	59.4 (7)
C6—O2—C7—N1	165.39 (18)	C18A—C19A—C20A—C16A	0.5 (11)
C6—O2—C7—C8	−14.3 (3)	C20A—C16A—C17A—Fe1	60.2 (9)
C6—C1—C9—C11	97.8 (2)	C20A—C16A—C17A—C18A	−1.0 (19)
C6—C1—C9—C8	−25.2 (2)		

### 2-Amino-4-ferrocenyl-5-oxo-5,6,7,8-tetrahydro-4H-chromene-3-carbonitrile monohydrate Hydrogen-bond geometry (Å, º)

*Cg*1 and *Cg*2 are the centroids of the Cp rings C16–C20 and C16*A*–C20*A*, respectively.

**Table:**

*D*—H···*A*	*D*—H	H···*A*	*D*···*A*	*D*—H···*A*
O3—H3*C*···O1	0.84 (1)	2.01 (1)	2.842 (2)	175 (3)
O3—H3*D*···N2^i^	0.83 (1)	2.28 (2)	3.011 (3)	147 (3)
N1—H1*A*···O3^ii^	0.86 (1)	2.01 (1)	2.859 (3)	168 (3)
N1—H1*B*···*Cg*1^iii^	0.86 (2)	2.86 (2)	3.696 (7)	164 (3)
N1—H1*B*···*Cg*2^iii^	0.84 (2)	2.86 (2)	3.677 (7)	165 (3)

Symmetry codes: (i) −*x*+2, −*y*+1, −*z*+1; (ii) *x*, *y*, *z*+1; (iii) *x*, *y*+1, *z*.
